# *Aeromonas salmonicida* subsp. *salmonicida* Early Infection and Immune Response of Atlantic Cod (*Gadus morhua* L.) Primary Macrophages

**DOI:** 10.3389/fimmu.2019.01237

**Published:** 2019-06-04

**Authors:** Manuel Soto-Dávila, Ahmed Hossain, Setu Chakraborty, Matthew L. Rise, Javier Santander

**Affiliations:** ^1^Marine Microbial Pathogenesis and Vaccinology Lab, Department of Ocean Sciences, Memorial University of Newfoundland, St. John's, NL, Canada; ^2^Department of Ocean Sciences, Ocean Science Centre, Memorial University of Newfoundland, St. John's, NL, Canada

**Keywords:** Atlantic cod, Gadiform, innate immunity, primary macrophages, *Aeromonas salmonicida*, Gram-negative

## Abstract

In contrast to other teleosts, Atlantic cod (*Gadus morhua*) has an expanded repertoire of MHC-I and TLR components, but lacks the MHC-II, the invariant chain/CD74, and CD4^+^ T cell response, essential for production of antibodies and prevention of bacterial infectious diseases. The mechanisms by which *G. morhua* fight bacterial infections are not well understood. *Aeromonas salmonicida* subsp. *salmonicida* is a recurrent pathogen in cultured and wild fish, and has been reported in Atlantic cod. Macrophages are some of the first responders to bacterial infection and the link between innate and adaptive immune response. Here, we evaluated the viability, reactive oxygen species (ROS) production, cell morphology, and gene expression of cod primary macrophages in response to *A. salmonicida* infection. We found that *A. salmonicida* infects cod primary macrophages without killing the cod cells. Likewise, infected Atlantic cod macrophages up-regulated key genes involved in the inflammatory response (e.g., *IL-1*β and *IL-8*) and bacterial recognition (e.g., *BPI/LBP*). Nevertheless, our results showed a down-regulation of genes related to antimicrobial peptide and ROS production, suggesting that *A. salmonicida* utilizes its virulence mechanisms to control and prevent macrophage anti-bacterial activity. Our results also indicate that Atlantic cod has a basal ROS production in non-infected cells, and this was not increased after contact with *A. salmonicida*. Transmission electron microscopy results showed that *A. salmonicida* was able to infect the macrophages in a high number, and release outer membrane vesicles (OMV) during intracellular infection. These results suggest that Atlantic cod macrophage innate immunity is able to detect *A. salmonicida* and trigger an anti-inflammatory response, however *A. salmonicida* controls the cell immune response to prevent bacterial clearance, during early infection.

## Introduction

Atlantic cod (*Gadus morhua*), one of the most important commercial fish species in the North Atlantic fisheries, has unusual modifications of the immune gene repertoire that set it apart from other teleosts (1). This Gadiform fish lacks the genes for the major histocompatibility complex class II (MHC-II), the invariant chain/CD74 (Ii), and CD4^+^ T cell response, representing an important evolutionary diversification of the adaptive immune system of vertebrates (2). The MHC-II binds antigens from extracellular pathogens, and the MHC-II-antigen complex activates helper CD4^+^ T cells, which play an essential role fighting bacterial infectious diseases (3).

The Atlantic cod appears to have compensated for the lack of the MHC-II pathway by expanding the number of MHC-I genes (4). This expanded MHC-I gene family has been divided into two clades, one maintaining the classical MHC-I functionality, and the other showing a MHC-II-like function (2). Indeed, around 80–100 copies of the MHC-I loci are found in the Atlantic cod genome, in contrast to other gadiformes that present only 40 copies (1, 2, 5), or to humans that harbor only ~10 copies (6). In addition to the MHC-I diversification, the Atlantic cod has expanded some Toll-like receptor (TLR) families, which have an important role in the innate immune response and pathogen detection (2, 7, 8). The Atlantic cod lacks TLR1, TLR2, and TLR5 that recognize bacterial surface antigens, however, this seems to be compensated by an expansion of the TLR7, TLR8, TLR9, and TLR22 families related to nucleic acids recognition (2, 6, 9, 10).

The Atlantic cod is a very successful teleost species and not particularly susceptible to infectious diseases (11), even though some of the prevalent marine bacterial pathogens such as *Vibrio anguillarum, Francisella noatunensis*, and *Aeromonas salmonicida* have been reported in wild and cultured cod (12–14).

*A. salmonicida*, is worldwide found in aquatic environments and have been implicated in the etiology of a large variety of fish diseases (15). *A. salmonicida* has five subspecies, *salmonicida, achromogenes, smithia, masoucida*, and *pectinolytica* (15). Immune response of Atlantic cod to subsp. *achromogenes* infection has been described (13, 16). In contrast, immune response of Atlantic cod to subsp. *salmonicida* has not been studied.

*Aeromonas salmonicida* subsp. *salmonicida* (hereafter *A. salmonicida*), a causative agent of the furunculosis, is a recurrent health problem for several marine fish species (17). This Gram negative, facultative anaerobic, non-motile, and bacillus shaped bacterium (18, 19), contains among others, a type-three secretion system that translocates to the eukaryotic cell several effector proteins, which influence immune response, including inflammation (20, 21).

Inflammation is a protective reaction of the host in response to bacterial infection, involving the migration of leukocytes, including macrophages, to the site of infection (22).

Macrophages, in addition to neutrophils, are the first defense line of vertebrates, including in Atlantic cod. Upon infection, macrophages are activated, secreting antimicrobial peptides (AMPs), cytokines, and chemokines, among others immune modulatory molecules (11, 23, 24). Activated macrophages have an increased phagocytic activity, correlated with an increased production of reactive oxygen species (ROS), and up-regulation of anti-bacterial gene transcription (25–27).

How Atlantic cod primary macrophages respond to *A. salmonicida* infection is unknown. Therefore, the aim of this study was to investigate the immune response of Atlantic cod head kidney primary macrophages to *A. salmonicida* infection.

We determined that Atlantic cod primary macrophages are able to mount an immune response and survive during the first 6 h of *A. salmonicida* infection. However, *A. salmonicida* invade the macrophages in a short period of time, remain in intracellular *A. salmonicida* containing vesicles, and modify macrophage immunity, likely preventing the action of AMPs and apoptosis.

## Materials and Methods

### *Aeromonas salmonicida* Growth Conditions

A single colony of *A. salmonicida* J223 (17) was grown routinely in 3 ml of Trypticase Soy Broth (TSB, Difco, Franklin Lakes, NJ) at 15°C in a 16 mm diameter glass tube and placed in a roller for 24 h. After growth, 300 μl of the overnight culture were added in 30 ml of TSB media using a 250 ml flask and incubated for 24 h at 15°C with aeration (180 rpm). The bacterial growth was monitored spectrophotometrically until O.D. 600 nm ~0.7 (1 × 10^8^ CFU ml^−1^) using the Genesys 10 UV spectrophotometer (Thermo Spectronic, Thermo Fischer Scientific Inc., Waltham, MA, USA). Then the bacterial culture was centrifuged at 6,000 rpm at room temperature for 10 min. The pellet was washed twice with PBS and centrifuged at 6,000 rpm at room temperature for 5 min, and finally resuspended in 300 μl of PBS (~5 × 10^10^ CFU ml^−1^). The concentrated bacterial inoculum was serial diluted and quantified by plating onto TSA supplemented with Congo red (50 μg ml^−1^).

### Formalin-Killed *A. salmonicida*

*A. salmonicida* J223 strain was grown in TSB media supplemented with 100 μM 2, 2′-dypyridyl at 15°C with aeration (180 rpm) up to an optical density of O.D. 600 nm ~0.7 (~1 × 10^8^ CFU ml^−1^). The bacterial cells were washed three times with PBS and then fixed with 6% formalin for 3 days at room temperature with gentile agitation. Formalin killed cells were dialyzed (Molecular Weight cut off 3.5 kDa; Spectra/Por, Laguna Hills, CA) in PBS three times and stored at 4°C at the concentration of 6 × 10^10^ CFU ml^−1^ until utilization.

### Fish Holding

Adult specimens of Atlantic cod 1.5 ± 0.2 kg (mean ± SE) were obtained from the Dr. Joe Brown Aquatic Research Building (JBARB) at the Department of Ocean Sciences, Memorial University of Newfoundland, Canada. The animals were kept in 21 m3 tanks, with flow-through (75.l × min^−1^) of sea water (6°C) and ambient photoperiod. The individuals were fed with commercial dry pellets (Skretting: 50% protein, 18% fat, 1.5% carbohydrate, 3% calcium, 1.4% phosphorus) with a ratio of 1% of body weight 3 days per week. The experiment was performed in accordance with the guidelines of the Canadian Council on Animal Care and approved by Memorial University of Newfoundland's Institutional Animal Care Committee (protocols #17-01-JS; #17-02-JS).

### Macrophage Isolation

Primary macrophages were isolated from Atlantic cod head kidney in accordance to the protocol established by Eslamloo et al. (28) with modifications. Briefly, head kidney tissues from six fish were aseptically removed and individually minced through 100 μm nylon sterile cell strainers (Fisher Scientific, Thermo Fisher Scientific, Waltham, MA, USA) in isolation media [(Leibovitz-15 (Gibco^®^, Gran Island, NY, USA) supplemented with 2 mM L-glutamine, 4.2 mM NaHCO_3_, 25 mM HEPES, 1.8 mM glucose, 20 U ml^−1^ heparin, 100 U ml^−1^ penicillin, 100 μg ml^−1^ streptomycin, and 1% Fetal Bovine Serum (FBS)]. After this period, 3 ml of cell suspension were centrifuged (400 × g at 4°C) for 40 min in a 25/51% Percoll gradient (GE Healthcare, Uppsala, Sweden). Macrophages collected from the macrophage-enriched interface were washed with phosphate buffered saline [PBS; 136 mM NaCl, 2.7 mM KCl, 10.1 mM Na_2_HPO_4_, 1.5 mM KH_2_PO_4_ (pH 7.2)] (29) twice and the number and viable cells were determined using the Countness™ cell counter (Invitrogen), and trypan blue stain (Invitrogen). After determining the numbers of cells from each sample, the primary macrophages were seeded in 22 mm 12-well or 35 mm 6-well cell-culture multidishes (Thermo Scientific, Roskilde, Denmark) at a concentration of 1 × 10^7^ cells ml^−1^. The plates were incubated at 15°C for 24 h in isolation media. After this period the cells were washed with PBS and incubated at 15°C for additional 24 h in 1 ml of culture media [Leibovitz-15 (Gibco^®^), supplemented with 2 mM L-glutamine, 4.2 mM NaHCO_3_, 25 mM HEPES, 1.8 mM glucose, 100 U ml^−1^ penicillin, 100 μg ml^−1^ streptomycin, and 1% FBS] to allow cell attachment until the infection assay.

### Gentamicin Exclusion Assay

Infections of primary macrophages with *A. salmonicida* were performed according to a previously established protocol (17). To remove the antibiotic present in the culture media, the isolated primary macrophages were washed once with 1 ml of PBS, and then inoculated with 1 ml of cultured media without antibiotics. After this, the primary macrophage monolayers were infected with 10 μL of bacterial suspension [~1 × 10^6^ cells ml^−1^; Multiplicity of Infection (MOI) 1:1 (bacteria:macrophage)] and incubated at 15°C. After 1 h post infection, the *A. salmonicida* attached to the Atlantic cod macrophages were quantified. The infected primary macrophage monolayers were washed 3 times with PBS, and lysed with 400 μl of Triton X100 (0.01%; Sigma) during 10 min (30) and then 600 μl of PBS were added to complete 1 ml of lysed macrophage suspension. Then the lysed macrophage suspensions were serially diluted (1:10) and plate/counted on TSA plates supplemented with Congo Red to determine the number of viable *A. salmonicida* per monolayer. The plates were incubated at 15°C for 5 days to determine the CFU per well. In addition, samples were taken for cell viability, RNA extraction, and transmission electron microscopy (see below for details).

For the invasion assay, cell monolayers were infected for 1 h, washed 3 times with PBS, followed by the addition of 1 ml of fresh culture media supplemented with gentamicin (10 μg ml^−1^, a higher concentration than the minimal inhibitory concentration for *A. salmonicida*) (31), and incubated at 15°C. Samples were taken at 2, 3, and 6 h post infection for bacterial count, cell viability, RNA extraction, and transmission electron microscopy. All the macrophages were isolated from three individual fish and triplicates were utilized for each treatment in the assays.

### Primary Macrophage Viability Determination

To determine the viability of infected primary macrophages, the cells were seeded in 12 wells plates, infected with *A. salmonicida*, and processed as described in the gentamicin exclusion assay. For each time point post *A. salmonicida* infection, the cells were washed with 1 ml of PBS and then treated with 500 μl of trypsin-EDTA (0.5%; Gibco) for 10 to 15 min. After this period, the trypsin was inactivated with 500 μl of culture media. The cells were stained with trypan blue (0.4%; Invitrogen) in a ratio of 1:1 (10 μl: 10 μl) and quantified using Countess™ Cell Counting Chamber Slides (Invitrogen) and Countess^®^ Automated Cell Counter (Invitrogen) according to the manufacturer's instructions. The numbers of alive and dead cells were determined at each time point post- infection. All the macrophages were isolated from three individual fish and technical triplicates were utilized in the assays.

### RNA Extraction and qPCR

To determine the effect of *A. salmonicida* on the innate immune response of Atlantic cod primary macrophages, samples of RNA were isolated from infected cells at 1, 2, and 6 h post *A. salmonicida* infection, using the previously described gentamycin exclusion methodology. Primary macrophages that were either mock infected with PBS or inoculated with 1 × 10^6^ CFU of formalin killed *A. salmonicida* were utilized as controls. Total RNA was extracted using TRIzol (Invitrogen), and purified using RNeasy (QIAGEN) following manufacturers' instruction (32). RNA samples were treated with TURBO DNA-free™ Kit (Invitrogen) for complete digestion of DNA and removal of remaining DNase and divalent cations, such as magnesium and calcium. Purified RNA samples were quantified and evaluated for purity using a Nano-quant spectrophotometer (Genway, UK), and evaluated for integrity by 1% agarose gel electrophoresis (29). cDNA was synthetized with the SuperScript™ III First-Strand Synthesis System (Invitrogen) using 500 ng of RNA per reaction and random hexamers according to the manufacturer's instructions.

Primer pair efficiencies were analyzed using a 20 ng μl^−1^ pooled cDNA from each set of samples, which was serially diluted (dilutions starting with 1 (20 ng μl^−1^), 1:3 (6.67 ng μl^−1^), 1:9 (2.22 ng μl ^−1^), 1:27 (0.74 ng μl^−1^), 1:81 (0.25 ng μl^−1^), 1:243 (0.08 ng μl^−1^), 1:729 (0.03 ng μl^−1^)). Primer pair efficiencies were calculated using the formula *E* = 10^(−1/slope)^ (33).

All qPCR reactions were done in a final volume of 20 μL, containing 10 μL of 1 × PowerUp-SYBR Master Mix (Applied BioSystems, Foster City, CA, USA), 1 μL (10 μM) of each primer, 6 μL of nuclease free water (Ambion), and 2 μL of cDNA. All samples were amplified and detected in a QuantStudio 3 (Applied BioSystems). The reaction mixtures were incubated for 2 min at 95°C, followed by 40 cycles of 1 s at 95°C, 30 s at 60°C, and finally 15 s at 95°C, 1 min at 60°C, and 15 s at 95°C. Initially, a total of five Atlantic cod genes were tested as reference gene candidates (EF-1α, ß-actin, Eif3, 18S, 60S). cDNA from a sub-set of samples (Supplementary Table 1) were utilized for evaluation of reference gene stability. The most stable gene for this set of individual samples was determined by using geNorm (M value 0.102) and BestKeeper (Value 0.101) (Supplementary Table 1). After this determination, the individual samples were analyzed. The mRNA gene expression was normalized to the Atlantic cod *elongation factor 1 alpha* (*EF-1a*) due to its stability across different treatments. Gene expression was determined using the comparative ^−ΔΔ*Ct*^ method (34).

The primers used in this study are listed in Table 1. In all cases, each qPCR was performed with triplicate samples and repeated with six independent fish.

**Table 1:** Primer sequences used in this experiment.

**Gene**	**Forward (5^′^to 3^′^)**	**Reverse (5^′^to 3^′^)**	**Tm^°^C**	**Efficiency (%)**	**References**
*IL-1b*	TGAGGACCTGCTCAACCTCT	TCTTCTGGTGGTCCCTCAAC	55.6	103.7	(35)
*IL-8*	GGTTTGTTCAATGATGGGCTGTT	GACCTTGCCTCCTCATGGTAATACT	56.5	98.4	(36)
*IL-10*	CCTATAAAGCCATCGGCGAGTTA	TGAAGTCGTCGTTTTGAACCAAG	56.6	100.1	(36)
*MHC-I*	CTAGCGTGGGACCTGAAGAC	CAGAGTGCTCTTCCCGTAGG	56.5	108.4	(35)
*g-type lysozyme*	CATTGACCAAGCCACTGGAATCCT	ATTCGACTCTACCGTCTCCAGTGT	59.3	102.3	(35)
*BPI/LBP*	GACCGTCAACGTGATGGCCCCGGT	CTTTGTTGGCCTCTATGCTGGAGAG	59.4	96.8	(37)
*Cathelicidin (CAMP)*	ATTGCAATTTCACCCTGAGC	CCAGACCTGCTCCTTCTCAC	56.4	108.1	(38)
*Transferrin*	GAGCTCCCATCGACAGCTAC	CAAACCCAGCAGAGGAGAAG	56.7	108.9	(39)
*Hepcidin (HAMP)*	CCACAGGCTCCTCTCAAGTC	CTGCAACTGCAATGCTGAAT	56.4	105.1	(38)
*nrf2*	TCGCAGTAGGAGCTGGATGA	CTCCGGTCTGTCCTTGGAAA	57.0	98.1	(40)
*nox1*	GCCTATATGATTGGCCTGATGAC	GCTGTGCTGAGTGGGTCGTA	55.3	108.6	(40)
*Mn-Sod*	ATGTGGCCTCCTCCATTGAA	GCATCACGCCACCTATGTCA	55.1	109.2	(40)
*Cu/Zn-Sod*	CATGGCTTCCACGTCCATG	CGTTTCCCAGGTCTCCAACAT	56.8	98.0	(40)
*cat*	GCCAAGTTGTTTGAGCACGTT	CTGGGATCACGCACCGTATC	57.3	101.0	(40)
*EF-1a*	GATGCACCACGAGTCTCTGA	GGGTGGTTCAGGATGATGAC	56.2	98.3	(35)

### Respiratory Burst Assay

Respiratory burst of primary cod macrophages infected with *A. salmonicida* was determined according to the protocol established by Smith et al. (41, 42) with some modifications. Briefly, isolated primary macrophages were infected with 10 μl of bacterial suspension (~1 × 10^6^ cells ml^−1^; MOI 1:1) and incubated at 15°C for 48 h. Primary macrophages inoculated with PBS were utilized as negative control, and phorbol myristate acetate (PMA 1 mM; Sigma) dissolved in dimethyl sulfoxide (DMSO) was utilized as positive control.

After 1 h post infection, cells were washed and the culture media was replaced with respiratory burst assay buffer (Leibovitz L-15 media supplemented with 1% BSA and 1 mM CaCl_2_). Then, 1 μl of dihydrorhodamine 123 (DHR, 5 mg/ml; Sigma) was diluted in 1 ml of PBS, and 50 μl of the dilution added to the macrophages for 15 min. Subsequently, 125 μl of PBS for negative control, or 125 μl of PMA (1 mM, final concentration 0.185 μM PMA) were added to the macrophages monolayers for 45 min to stimulate ROS production (43, 44). Finally, the macrophages were detached using 1 ml of trypsin-EDTA (0.5%; Gibco), washed with PBS, centrifuged for 5 min (500 × g at 4°C), and resuspended in fluorescence-activated cell sorting (FACS) buffer (1% FBS in PBS). Fluorescence was detected from 10,000 cells using a BD FACS Aria II flow cytometer (Becton Dickinson™) and analyzed using BD FACS Diva v7.0 software (BD Biosciences, San Jose, CA, USA). The PBS control cells were used to define the region of ROS negative cells, and based on this gating the FITC positive cells were identified. The mean fluorescence intensity and percentage of FITC-positive cells were determined for each condition. The experiments were conducted in macrophages isolated from six independent fish, and 10,000 events were measured for each sample.

### Transmission Electron Microscopy (TEM)

Primary macrophages were fixed in anhydrous paraformaldehyde (4%; Electron Microscope Sciences, Hatfield, PA, USA) at 4°C until the samples were processed at the Electron Microscopy/Flow Cytometry Unit at Memorial University of Newfoundland. The cells were pelleted and resuspended in Karnovsky fixative for 20 min (45), washed in 0.1 M sodium cacodylate buffer pH 7.4 for 5 min, and post-fixed in 1% Osmium tetroxide during 15 min. After this, the fixed cells were dehydrated in increasing concentrations of ethanol and acetone followed by infiltration with EPON resin (Sigma). Cells were pelletized between incubation steps. Resin blocks were polymerized in BEEM capsules (Electron Microscope Sciences) overnight at 70°C and ultra-thin sections were cut with a diamond knife (Diatome, Hatfield, PA, USA). The ultra-thin sections were mounted on 300 copper mesh grids, stained with uranyl acetate and lead citrate, and examined in a Tecnai™ Spirit TMA with an accelerating voltage of 80 kV. Cells incubated for 3 h with PBS (control), J223 strain, and formalin-killed *A. salmonicida* were observed.

### Statistical Analysis

All data are shown as the mean ± standard error (SE). Assumptions of normality and homogeneity were tested for the detected variances. A Kruskal-Wallis nonparametric test was performed for gentamicin exclusion assay results. Gene expression and ROS data were analyzed using a repeated measures two-way ANOVA test, followed by Sidak multiple comparisons *post hoc* test to identify significant differences of each treatment in different times and between treatments in the same time point. Differences were considered significant at *P* < 0.05. All statistical analyses were performed using GraphPad Prism (GraphPad Software, La Jolla California USA, www.graphpad.com).

## Results

### Macrophage Viability and *Aeromonas salmonicida* Infection

**Figure 1:**
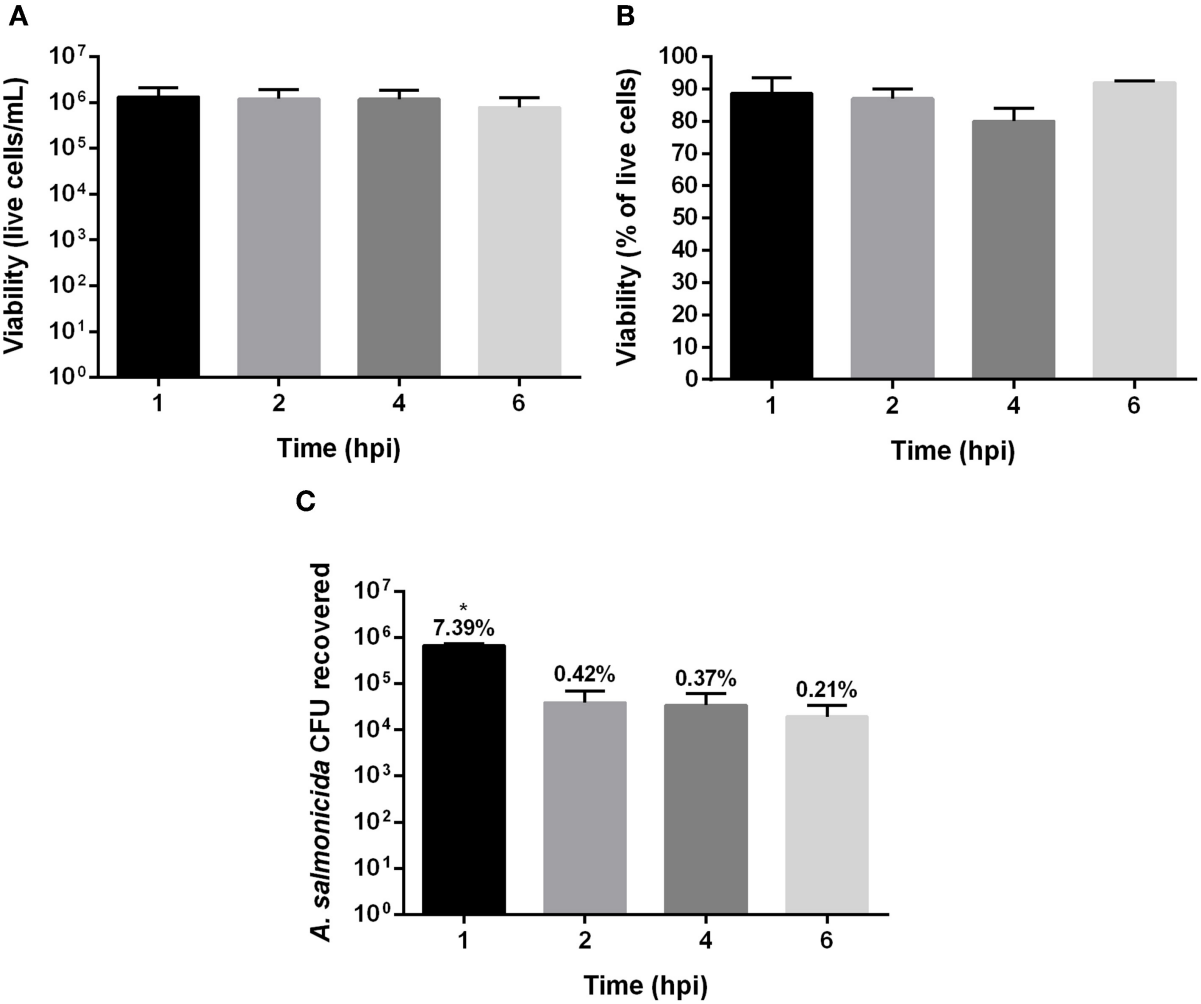
Gentamicin exclusion assay in Atlantic cod macrophages infected with *Aeromonas salmonicida* subsp. *salmonicida*. **(A)** Live cells **(A)** and **(B)** percentage of viability, after 1, 2, 4, and 6 h post infection. **(C)** Colony forming unit (CFU) recovered after 1, 2, 4, and 6 h post infection with *A. salmonicida*. The percentage showed above bars indicate the total % of attachment (1 h post infection) and invasion (2, 4, and 6 h post invasion) of *A. salmonicida* in Atlantic cod macrophages, *p* < 0.005. Each value represents the ± S.E.M (*n* = 3). Symbol (^*^) indicate statistical differences between each time post infection.

The viability of Atlantic cod primary macrophages infected with *A. salmonicida* was determined at 1, 2, 4, and 6 h post-infection. The results did not show significant differences between the time points post-infection in the number of live cells and percentage of viability. For instance, after 1, 2, 4, and 6 h post-infection 1.32 × 106 ± 8.02 × 105, 1.18 × 106 ± 7.13 × 105, 1.17 × 106 ± 6.64 × 105, and 7.77 × 105 ± 4.77 × 105 cells were quantified, respectively (Figures 1A,B). The percentage of viability during the infection process also did not show significant differences between time points post-infection. After 1, 2, 4, and 6 h the infected cells showed a viability of 89% ± 4.8, 87% ± 3.1, 80% ± 4.2%, and 92% ± 6%, respectively (Figure 1B).

Although the primary cod macrophage cells seemed to survive the *A. salmonicida* infection, the bacteria infected and invaded the cell monolayers. The Atlantic cod macrophages were infected with a MOI of 1:1 (bacteria: macrophage) with an initial inoculum of 9.6 × 106 CFU. After 1 h post-infection, 7.39% (6.8 × 105 CFU) was attached to the macrophage monolayer, and after 2, 4, and 6 h post-infection, 0.42% (3.87 × 104 CFU), 0.37% (3.42 × 104 CFU), and 0.21% (1.94 × 104 CFU) of *A. salmonicida* were intracellularly located, respectively (Figure 1C).

### Gene Expression Response of Atlantic Cod Macrophages to *A. salmonicida* Infection

**Figure 2:**
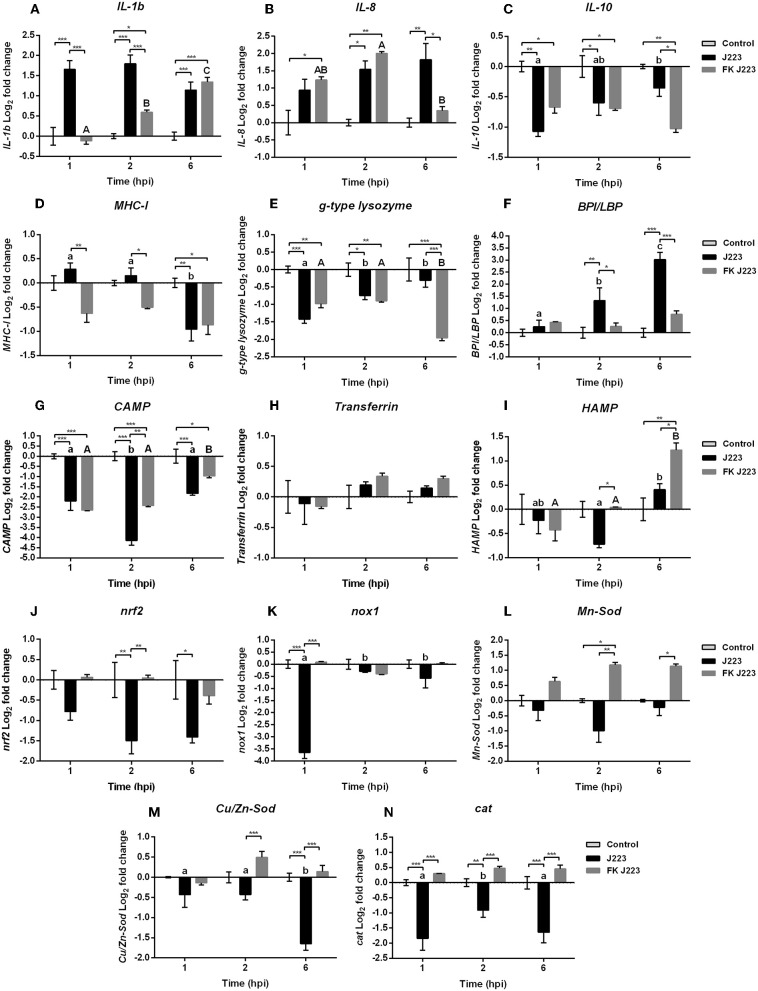
Gene expression of **(A)** Interleukin 1b (*IL-1b*), **(B)** Interleukin 8 (*IL-8*), **(C)** Interleukin 10 (*IL-10*), **(D)** Major histocompatibility complex class 1 (*MHC-I*), **(E)** Goose type lysozyme (*g-type lysozyme*), **(F)** Bactericidal permeability increasing protein / lipopolysaccharide-binding protein (*BPL/LBP*), **(G)** Cathelicidin (*CAMP*), **(H)** *Transferrin*, **(I)** Hepcidin (*HAMP*), **(J)** Nuclear factor erythroid 2-related factor 2 (*nrf2*), **(K)** NADPH oxidase 1 (*nox1*), **(L)** Mn superoxide dismutase (*Mn-Sod*), **(M)** CuZn superoxide dismutase (*Cu/Zn-So*d), and **(N)** Catalase (*cat*) in Atlantic cod primary macrophages isolated from head kidney and infected with live (J223) and formalin-killed *A. salmonicida* (FK 223) at different times post infection (1, 2, and 6 h). Relative expression was calculated using the 2(^−ΔΔ*Ct*^) method and Log2 converted using *EF-1a* as internal reference gene. Different letters represent significant differences between primary macrophages infected with J223 strain (lower case) or inoculated with FK J223 (upper case) at different times-points. Asterisks (^*^) represent significant differences between treatments on each time-point (^*^p < 0.05, ^**^p < 0.01, ^***^p < 0.001). Each value is the mean ± S.E.M (*n* = 6).

Quantitative real-time expression of selected genes related to Atlantic cod macrophage immunity was evaluated during *A. salmonicida* infection, and compared with macrophages inoculated with inactivated *A. salmonicida* (formalin-killed), and PBS inoculated controls. Significant increases in the expression of the pro-inflammatory cytokine interleukin 1β (*IL-1*β) gene were observed 1, 2, and 6 h post *A. salmonicida* infection compared to the time-matched PBS controls (Figure 2A). An up-regulation of the pro-inflammatory cytokine interleukin 8 (*IL-8*) gene, was also observed, nonetheless, this up-regulation occurred at 2 h, and 6 h post *A. salmonicida* infection compared to the PBS controls (Figure 2B). In contrast, the macrophages inoculated with inactivated *A. salmonicida* showed a higher expression of *IL-1*β at 2 and 6 h post-inoculation compared to their respective PBS controls (Figure 2A). *IL-8* was up-regulated in cells treated with formalin-killed bacteria after 1 and 2 h post-inoculation (Figure 2B). At 6 h post-inoculation with the inactivated *A. salmonicida*, the expression of *IL-8* in cod macrophages did not show differences compared to the PBS inoculated cells (Figure 2B). The expression of *IL-1b* was significantly up-regulated at 1 and 2 h after *A. salmonicida* infection compared with bacterin-exposed macrophages, whereas for *IL-8* significant up-regulation in infected vs. bacterin-exposed macrophages was only seen at the 6 h time point (Figures 2A,B).

In contrast, the relative expression of the anti-inflammatory cytokine Interleukin 10 (*IL-10*) gene, was significantly down-regulated after 1 and 2 h post *A. salmonicida* infection compared to the non-infected control macrophages (Figure 2C). Cod macrophages inoculated with the inactivated pathogen also showed a significant down-regulation at 1, 2, and 6 h compared with PBS controls (Figure 2C).

Genes involved in antigen recognition and host defense showed different patterns of expression after macrophage exposure to the live or inactivated *A. salmonicida*. In the case of the major histocompatibility complex class I (*MHC-I*) gene, a significant down-regulation was observed after 6 h post live *A. salmonicida* infection and post-inoculation with the formalin-killed bacteria (Figure 2D).

A down-regulation of the relative expression of the Goose-type lysozyme (*g-type lysozyme*) gene was observed at 1 and 2 h post *A. salmonicida* infection, and at 1, 2, and 6 h post inoculation with the formalin-killed pathogen, compared to their respective controls (Figure 2E). At 6 h post-treatment, g-type lysozyme expression was significantly different in infected vs. inactivated pathogen exposed macrophages (Figure 2E).

In contrast, the bactericidal permeability-increasing protein/lipopolysaccharide-binding protein (*BPI/LBP*) gene, involved in the antimicrobial defense against Gram negative bacteria, showed a significant up-regulation at 2 and 6 h post-infection only in cells inoculated with the live bacteria compared with the control and formalin-killed inoculated treatments (Figure 2F).

The relative expression of AMPs encoding genes showed different expression patterns. Cathelicidin (*CAMP*) gene was down-regulated in macrophages infected with *A. salmonicida* and those inoculated with formalin-killed *A. salmonicida* at 1, 2, and 6 h post-infection compared with time-matched PBS controls (Figure 2G). Macrophages infected with *A. salmonicida* showed a significant down-regulation of *CAMP* 2 h post-infection compared to the cells inoculated with inactivated bacteria (Figure 2G).

*Transferrin* did not show variation in the level of expression of both treatments compared with the time-PBS matched controls during the assays, as well as, between macrophages inoculated with live and bacterin *A. salmonicida* (Figure 2H). Hepcidin (*HAMP*) expression was significantly up-regulated only at 6 h post-exposure to the formalin-killed *A. salmonicida* (Figure 2I). However, *HAMP* was significantly down-regulated at 2 h post-infection with *A. salmonicida* (Figure 2I).

Expression of genes involved in the synthesis of ROS was also evaluated (Figures 2J–N). Nuclear factor erythroid 2-related factor 2 (*nrf2*), a transcriptional factor that is translocated into the nucleus under oxidative stress and initiates transcription of antioxidative genes (40), did not show transcriptional variation in cells inoculated with formalin-killed *A. salmonicida* compared to their respective controls (Figure 2J). In contrast, macrophages infected with *A. salmonicida* showed a significant down-regulation in the expression of *nrf2* at 2 and 6 h post-infection (Figure 2J). After 1 h of infection, *nrf2* transcript was significantly lower expressed in pathogen-infected macrophages compared to bacterin-exposed macrophages.

The expression of NADPH oxidase 1 (*nox1*) gene, which encodes a membrane-bound pro-oxidant enzyme that catalyzes superoxide synthesis, was significantly down-regulated 1 h post-infection with *A. salmonicida* compared to the non-treated cells and the bacterin-exposed macrophages (Figure 2K). Furthermore, this gene was significantly lower expressed at 1 h compared with 2 and 6 h post-infection (Figure 2K).

The relative expression of the Mn superoxide dismutase (*Mn-Sod*) and the CuZn superoxide dismutase (*Cu/Zn-Sod*) genes, involved in the transformation of superoxide into H2O2 in the mitochondria and the cytosol, respectively, also was evaluated. The *Mn-Sod* was significantly up-regulated at 2 h post-inoculation with inactivated *A. salmonicida*, compared to the controls (Figure 2L). In contrast, macrophages infected with *A. salmonicida* showed a *Mn-Sod* down-regulation tendency, with a significant down-regulation at 2 and 6 h post-infection compared to the bacterin-exposed macrophages (Figure 2L).

Similar patterns were observed in *Cu/Zn-Sod* and *catalase* (*cat*) expression, where a higher expression was observed post-inoculation with the formalin-killed *A. salmonicida* and a down-regulation was observed in cells infected with *A. salmonicida* (Figures 2M,N). For instance, *Cu/Zn-Sod* show a significant down-regulation at 6 h post *A. salmonicida* infection compared to the PBS control inoculated cells, and 2 and 6 h post-infection compared to cells treated with the inactivated pathogen (Figure 2M).

The relative expression of *cat*, which encodes for the catalase enzyme that plays an important role in H_2_O_2_ detoxification (40), showed a down-regulation at 1, 2, and 6 h in macrophages infected with *A. salmonicida* compared to the control and the cells treated with the inactivated pathogen (Figure 2N). In contrast, macrophages exposed to inactivated *A. salmonicida* did not showed significant differential expression of *cat*, compared to their respective controls (Figure 2N).

### Reactive Oxygen Species (ROS) Production

**Figure 3:**
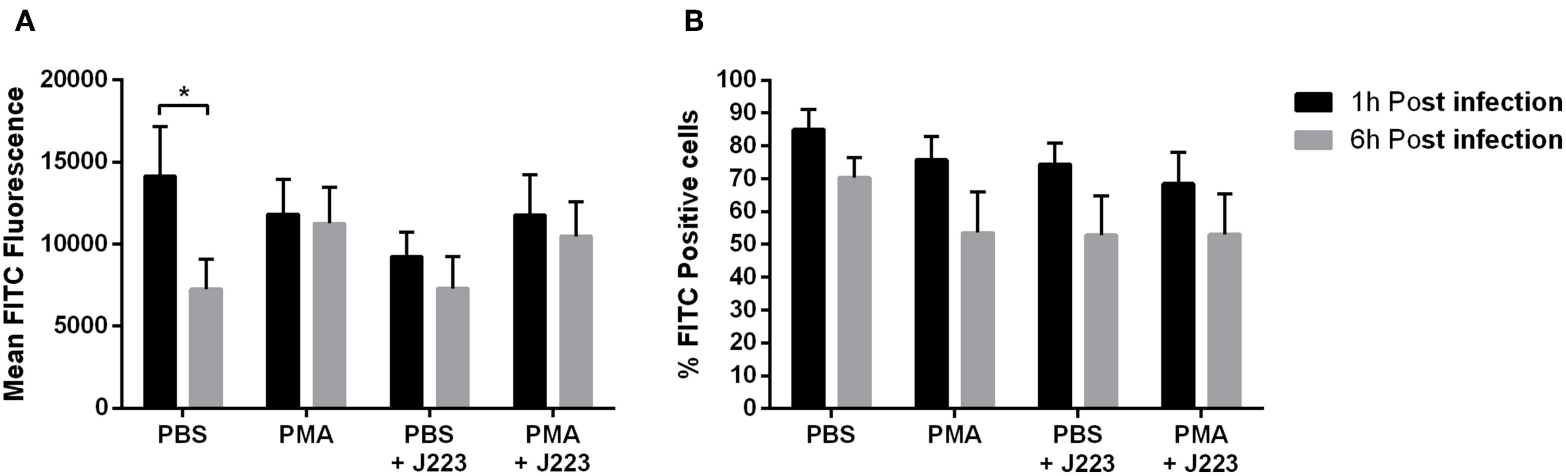
Reactive oxygen species (ROS) production in Atlantic cod macrophages after 1 and 6 h post-infection with *A. salmonicida*. **(A)** Mean FITC fluorescence and **(B)** percentage of FITC positive cells were obtained by flow cytometry. PBS inoculated cells (PBS) and PMA inoculated cells were utilized as negative and positive controls, respectively. Each value is the mean ± S.E.M (*n* = 6). Symbol (^*^) indicate differences on each group at different times of infection, *p* < 0.005.

ROS production was determined in Atlantic cod primary macrophages infected with *A. salmonicida*. Macrophages inoculated with PBS or PMA were utilized as negative and positive controls, respectively. ROS production was analyzed at 1 and 6 h post *A. salmonicida* infection. We did not observe significant differences in ROS production between treatments. Macrophages treated with PBS showed that 85.1% ± 6.1 and 70.4% ± 6.2 were producing ROS after 1 and 6 h, respectively. Macrophages treated with PMA showed that 75.8% ± 7.0 and 53.6% ± 12.4 were producing ROS after 1 and 6 h, respectively. Similarly, macrophages treated with PBS and infected with *A. salmonicida* showed that 74.5% ± 6.4 and 52.9% ± 11.9 of the cells were producing ROS after 1 and 6 h post infection, respectively. Additionally, macrophages treated with PMA and infected with *A. salmonicida* showed that 68.5% ± 9.5 and 53.1% ± 12.4 of the cells were producing ROS after 1 and 6 h post treatment, respectively (Figures 3A,B).

### Transmission Electron Microscopy (TEM)

**Figure 4:**
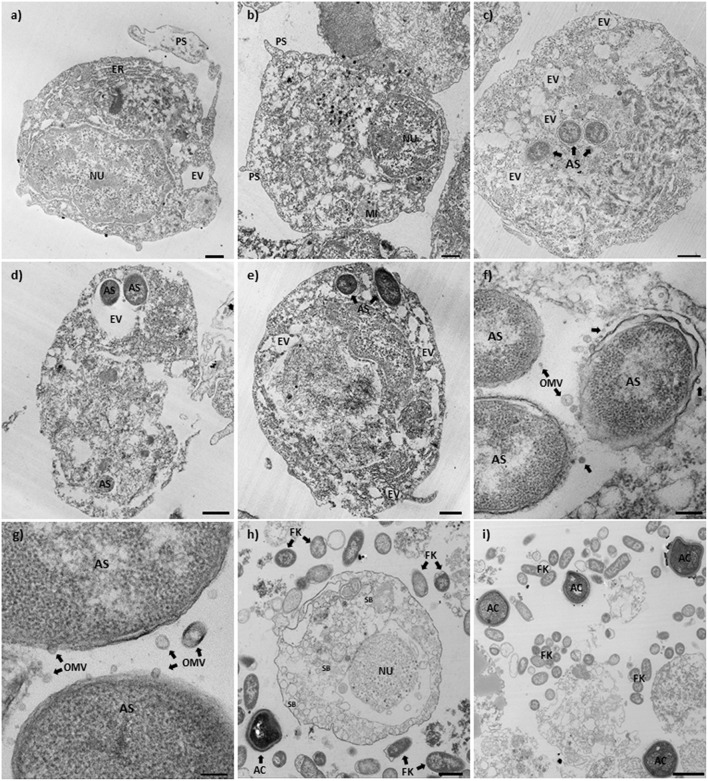
Transmission electron microscopy of Atlantic cod macrophages infected with *A. salmonicida*. **(a,b)** Mock infected Atlantic cod head kidney macrophages (control). (× 2,100, scale bar 1 μm). **(c–e)** Atlantic cod head kidney macrophages infected with *A. salmonicida*. (c and d: × 2700, scale bar 1 μm; e: × 2,100, scale bar 1 μm). **(f,g)** Intracellular *A. salmonicida*. (f: × 15,000, scale bar 200 nm; g: × 30,000, scale bar 100 nm). **(h,i)** Atlantic cod head kidney macrophages infected with the formalin killed *A. salmonicida*. (h: × 2,700, scale bar 1 μm; i: × 1,650, scale bar 2 μm). NU, Nucleus; PS, Pseudopodia; ER, Endoplasmic reticulum; MI, Mitochondria; AS, *A. salmonicida*; EV, Endoplasmic vesicles; OMV, Outer membrane vesicles (arrows); FK, Formalin killed *A. salmonicida*; SB, Secretion bodies; and AC, Apoptotic bodies.

Cod primary macrophages infected with *A. salmonicida* were visualized 3 h post-infection using TEM. A group of non-infected cells were utilized as reference control (Figures 4a,b). These cells showed a rounded cell morphology, large nucleus, and evident presence of cell organelles (e.g., mitochondria, endoplasmic reticulum, endocytic vesicles) and pseudopodias (Figures 4a,b). In contrast, Atlantic cod macrophages infected with *A. salmonicida* showed poorly defined nuclei, membrane ruffling, and large vesicles containing *A. salmonicida* cells (average of 2–3 bacterial cells per macrophage, a maximum of 8 bacterial cells per macrophages, and 70–80% macrophages infected) (Figures 4c–e). Furthermore, secretion of *A. salmonicida* outer membrane vesicles (OMVs) was observed in intracellular bacterial cells (Figures 4f,g).

Macrophages inoculated with the formalin killed *A. salmonicida* showed a defined nucleus and a large number of secretion bodies within the cytoplasm (Figure 4h). Apoptotic-like bodies were observed in high numbers in the presence of extracellular *A. salmonicida* bacterin (Figure 4i).

## Discussion

Atlantic cod lacks the genes for MHC-II, the invariant chain/CD74 (Ii), and CD4+ T cell response, representing a paradigm in the context of adaptive immunity against bacterial infectious diseases (2, 3). Additionally, Atlantic cod lacks TLR1, TLR2, and TLR5 that recognize bacterial surface antigens (2, 6, 9, 10). However, this absence seems to be compensated for by an expansion of the MHC-I (1, 4, 5) and the TLR7, TLR8, TLR9, and TLR22 families (2, 8). Nevertheless, how Atlantic cod fight bacterial infections is unknown. Here, we evaluated the early response of Atlantic cod primary macrophages to *A. salmonicida* infection.

To evaluate the early response of Atlantic cod primary macrophages to *A. salmonicida* infection, a gentamicin exclusion assay was conducted. The macrophage viability results obtained 1 h post infection (attachment) and 2, 4, and 6 h post-infection (invasion) showed similar viability, with 89% ± 4.8, 87% ± 3.1, 80% ± 4.2%, and 92% ± 6%, respectively (Figure 1B). In contrast, Atlantic salmon primary macrophages isolated and infected under similar conditions, showed 3 h post *A. salmonicida* infection a viability around ~40% (Supplementary Data and Supplementary Figures 1A,B). Similar reduction in viability was reported in non-phagocytic Chinook salmon embryo cell line (CHSE-214) infected with the *A. salmonicida* J223 strain, showing a viability of ~40% at 2 and 4 h post-infection (17). These results reveal that Atlantic cod macrophages are more resistant to *A. salmonicida* infection compared with Atlantic salmon macrophages and CHSE-214 cells.

The attachment of *A. salmonicida* in cod primary macrophages was 7.39%, and only 0.42, 0.37, and 0.21% was able to invade 2, 4, and 6 h post-infection, respectively (Figure 1C). The study conducted in CHSE-214 embryo cell line infected with *A. salmonicida* J223 strain showed an attachment of ~60% at 1 h post-infection, and an invasion of 0.47% and 0.29% after 2 and 4 h post-infection, respectively (17). Moreover, *A. hydrophila* isolated from ornamental fish, showed an attachment between 75 and 80% in the mammalian cell line CaCo-2 (cells from human colon adenocarcinoma) at 1 h post-infection (46). In contrast, the attachment of *A. salmonicida* J223 in Atlantic salmon primary macrophages was 10.7% at 1 h post-infection, and the invasion was 0.42, 0.26, and 0.28% after 2, 3, and 4 h post-infection, respectively (Supplementary Figure 1C). These results suggest that primary macrophages are less susceptible to be infected by *A. salmonicida* than non-phagocytic cells, even when the intracellular *A. salmonicida* recovered in non-phagocytic and phagocytic cells have shown to be relatively similar.

The transcriptional profiles of cytokine genes, antibacterial genes, antimicrobial peptide genes, and ROS related genes were determined using qPCR. The observed up-regulation of *IL-1*β and *IL-8* after infection with *A. salmonicida* or inoculation with formalin killed *A. salmonicida* (Figures 2A,B) indicates a canonical macrophage innate immune response (24, 47). Coincident with the up-regulation of the pro-inflammatory *IL-1*β and *IL-8* genes (Figures 2A,B), the anti-inflammatory *IL-10* gene was down-regulated in macrophages infected with *A. salmonicida* or inoculated with the formalin killed pathogen (Figure 2C).

Similar up-regulation of the pro-inflammatory cytokine *IL-1*β was observed in Atlantic cod intramuscular injected with *A. salmonicida* subsp. *achromogenes*, meanwhile an up-regulation in both, *IL-1*β and *IL-8*, has been reported in Atlantic cod gill epithelial cells infected with *Vibrio anguillarum* and *A. salmonicida* (48), and in Atlantic cod macrophages infected with *Francisella noatunensis* (49), reinforcing the importance of these canonical interleukins against Gram-negative pathogens during the first hours of infection.

Antigen recognition and host defense genes, like *MHC-I, g-type lysozyme*, and *BPI/LBP* were also evaluated. As mentioned previously, BPI/LBP participates in the recognition of lipopolysaccharide (LPS) (50, 51), the major component of Gram negative bacterial outer membrane (52), MHC-I participate in the recognition of intracellular pathogens, like viruses or cytoplasmic invader bacteria (3), and *g-type lysozyme* is related to both Gram positive and Gram negative antibacterial activity (53). Interestingly, *BPI/LBP* was up-regulated only after infection with live *A. salmonicida* but not in presence of formalin-killed *A. salmonicida* (Figure 2F). *BPI/LBP* up-regulation has been observed in Atlantic cod vaccinated with inactivated *V. anguillarum* and *A. salmonicida* (11, 51), and in Atlantic cod intestinal epithelial cells after exposure with the bacterial probiotics GP21 (*Pseudomonas* spp.) and GP12 (*Psychrobacter* spp.) isolated from the intestinal tract of an adult Atlantic cod (54, 55).

The AMP-encoding genes *CAMP* and *transferrin*, both involved in the iron homeostasis, showed unexpected transcriptional profiles (Figures 2G,H), meanwhile *HAMP*, also related to the iron ion homeostasis, was the only AMP-encoding gene that showed an up-regulation after inoculation with the inactivated bacteria (Figure 2I). *CAMP* was down-regulated after infection with the live bacteria or inoculation with the formalin-killed pathogen (Figure 2G), meanwhile *transferrin* did not show variations compared to the controls (Figures 2H,I). In the case of *HAMP*, up-regulation was observed only at 6 h post-inoculation with the inactivated pathogen (Figure 2I). As previously mentioned, it has been reported that *CAMP, transferrin*, and *HAMP* AMPs are key during bacterial infection, and in general these genes are expressed in several immune tissues of Atlantic cod after bacterial (e.g., *Mycobacterium chelonei, Aeromonas salmonicida* subsp. *salmonicida*, and *Aeromonas salmonicida* subsp. *achromogenes*) and viral (infectious pancreatic necrosis virus) infection, or viral mimic [poly (I:C)] stimulation (11, 36, 56). However, according to our results, these genes were either lower expressed (*CAMP* and *HAMP*) or not affected (*transferrin*) in Atlantic cod macrophages compared with formalin-killed *A. salmonicida* stimulation (Figures 2G–I). A study in Atlantic cod intramuscular infected with *A. salmonicida* subsp. *achromogenes* showed an up-regulation in the expression of *transferrin* and *HAMP* (16). In contrast, we found that in Atlantic cod primary macrophages, the infection with *A. salmonicida* subsp. *salmonicida* down-regulated the expression of *CAMP* and *HAMP*. This can suggest a different mechanism of infection between *A. salmonicida* subsps.

The Atlantic cod *CAMP* has a potent antimicrobial activity against Gram-negative bacteria and fungi (57). Nonetheless, some bacterial pathogens, like *V. anguillarum, A. salmonicida* subsp. *achromogenes* and *A. hydrophila* are able to evade the action of *CAMP* (57), and this can be the case for *A. salmonicida* J223 in Atlantic cod macrophages.

Atlantic cod injected with turpentine oil, an inducer of acute immune response that involves inflammation and other biological processes (e.g., hemostasis), showed an increase in the relative expression of these AMP genes after 24 h of injection (39), and similar results were observed in intestinal epithelial cells after probiotic exposure to the probiotics GP21 and GP12 (55). Moreover, a study conducted in Atlantic cod stimulated with formalin-killed *A. salmonicida* showed lower levels of expression in the transcripts encoding *CAMP* and *HAMP* in head kidney and spleen, compare with PBS control samples (38). In this study, only a pick was observed after 24 h of stimulation with the inactivated pathogen (38). Therefore, our results suggest that likely more time is required for Atlantic cod macrophages to up-regulate the *CAMP, transferrin*, and *HAMP* genes after inactivated *A. salmonicida* exposition.

A study conducted in the Gram-negative bacteria, *Pseudomonas syringae*, showed that outer membrane vesicles (OMVs) can potentially suppress the action of AMPs (58). OMVs bind and sequester AMPs to prevent bacterial cell damage, and also induce the release of peptidases, proteases, and other lytic enzymes to degrade the host AMPs (58, 59). Combining this reported evidence with our results, where a high number of OMVs were released by *A. salmonicida* during intracellular infection (Figures 4f,g), we hypothesize that the presence of OMVs during intracellular infection might be related to a mechanism by which the bacterium tolerates the action of host AMPs or translocate virulence factors to the host cell. This is in addition to unknown *A. salmonicida* mechanism involving the down-regulation of AMP-related genes.

Typically, macrophages phagocytize the invading bacteria, assemble the lysosome, and eliminate them through the action of several enzymes and production of ROS (40). Here, we evaluated the expression of *nrf2, nox1, Mn-Sod, Cu/Zn-Sod*, and *cat* genes that are part of the redox system and antioxidant enzymes related to ROS synthesis (40, 60). Relative expression levels of *nrf2, nox1, Mn-Sod, Cu/Zn-Sod*, and *cat* genes in cod primary macrophages were down-regulated after infection with *A. salmonicida* (Figures 2J–N). In contrast, macrophages inoculated with the formalin-killed *A. salmonicida*, showed an up-regulation in the expression of the *Mn-Sod* and *Cu/Zn-Sod* genes (Figures 2L–N).

The macrophage gene expression of *nrf2, nox1, Mn-Sod, Cu/Zn-Sod*, and *cat* after *A. salmonicida* infection, together with the ROS flow cytometry results, shows that Atlantic cod macrophages do not increase ROS levels after exposure to *A. salmonicida* (Figures 3A,B).

High basal levels of ROS production have been observed in non-induced Atlantic cod blood phagocytes (43), suggesting that ROS synthesis in Atlantic cod cells is related to the *in vitro* culture conditions (43). However, we hypothesize that high basal production of ROS could be normal in *G. morhua*, and the lack of ROS production, above the basal levels after *A. salmonicida* infection, could be associated to mechanisms used by *A. salmonicida* to control the macrophage immune response, as described previously in other pathogens such as *Mycobacterium leprae, R. salmoninarum, Salmonella* spp, and *Edwardsiella tarda* (61–64).

Macrophages are usually highly efficient killers of bacteria, however pathogenic bacteria, like *A. salmonicida*, have evolved multiple strategies to infect, avoid enzymatic digestion, and trigger immunosuppression of the host (49). Our TEM results showed that *A. salmonicida* was localized intracellularly in bacteria containing vesicles (Figures 4c,e). Also, we observed that the cytoskeleton of the infected cells was rearranged, and several structures, like the nuclei, pseudopodia, and endoplasmic reticulum, were not observed in infected macrophages, in contrast to non-infected cells (Figures 4a–e). Additionally, the TEM images of infected macrophages showed a significant number of intracellular bacteria at 3 h post-infection (Figures 4c–e). Not all Gram-negative pathogens gain access to macrophages in high numbers. For instance, *F. noatunensis* and *F. tularensis* invade in lower numbers, even 24 h post-infection with high infectious doses. Thus, few *F. tularensis* cells are required to cause fatal diseases (49, 65–68). In contrast to these previous studies, our results indicate that *A. salmonicida* required a higher number of infecting bacterial cells, compared to other bacterial pathogens, to have a productive infection (Figures 4c–e), Additionally, a single macrophage is infected with a significant number of bacterial cells (maximum of 8 bacterial cells per macrophage was founded, 2–3 average) suggesting that these are the target cells of *A. salmonicida*.

An interesting finding of our study was the presence of several OMVs produced by *A. salmonicida* during intracellular infection (Figures 4f,g). The OMVs are virulence factors released by mammalian pathogens like *Neisseria meningitides, Escherichia coli, Vibrio* spp., *Brucella* spp., among others (69). These OMVs play an important role during pathogenesis, delivering toxins and immunomodulatory proteins to the host cell (69). Also, OMVs have been observed and described previously in marine pathogens such as *F. noatunensis* subsp. *orientalis, Edwardsiella anguillarum*, and *Piscirickettsia salmonis* (70–72). Our results suggest that *A. salmonicida* release the OMVs during intracellular infection in order to control the immune response of the Atlantic cod primary macrophages, and perhaps neutralizing antimicrobial peptides to avoid lysis.

Phagocytosis is a highly efficient mechanism for bacterial elimination used by macrophages. However, Atlantic cod macrophages exposed to formalin-killed *A. salmonicida* for 3 h showed that most of the formalin-killed bacteria were localized in the extracellular milieu, with a reduced number of bacterial cells in phagocytic vesicles (Figures 4h,i). This results suggest that *A. salmonicida* promote macrophage phagocytosis, in contrast to inactivated bacteria.

Also, we observed that Atlantic cod macrophages exposed to formalin-killed *A. salmonicida* produced a large amount of apoptotic-like bodies (Figures 4h,i). Programmed cell death (i.e., apoptosis), is a highly regulated process and an important mechanism used by the host to prevent infectious diseases (73). Cells undergoing apoptosis maintain membrane integrity until very late in the process, unlike cells undergoing necrosis, but produce several morphological and biochemical changes inside the cells, including chromatin condensation, nuclear segmentation, internucleosomal DNA fragmentation, and cytoplasmic vacuolization (74, 75). Similar morphological changes were observed in Atlantic cod macrophages inoculated with formalin-killed *A. salmonicida* (Figures 4h,i). This suggest that *A. salmonicida* is displaying pathogenesis mechanisms to avoid not only phagocytosis but also to prevent apoptosis.

## Conclusion

In this study we evaluated the infection of Atlantic cod macrophages with *A. salmonicida* J223 strain. *A. salmonicida* infects and invades Atlantic cod primary macrophages. We found between 2 and 8 *A. salmonicida* cells per infected macrophage. The infected Atlantic cod macrophages survived during the first 6 h of *A. salmonicida* infection. Nevertheless, TEM observations showed that *A. salmonicida* remained in *A. salmonicida*-containing vesicles. Gene expression results from infected macrophages suggest that *A. salmonicida* modulate the expression of several genes involved in the innate immune response. For instance, relative expression of *HAMP, nrf2, nox1, Mn-Sod, Cu/Zn-Sod*, and *cat* genes were down-regulated and *BPI/LBP* was up-regulated after *A. salmonicida* infection. Additionally, we observed that *A. salmonicida* secrete OMVs during intracellular infection. These results suggest that *A. salmonicida* has immune suppressive mechanisms to control cod macrophage immune response, where OMVs could play an essential role.

In contrast, macrophages inoculated with formalin-killed *A. salmonicida*, showed a canonical innate immune response, where most of the evaluated genes were up-regulated or not induced, like the relative expression of *HAMP, nrf2, nox1, Mn-Sod, Cu/Zn-Sod*, and *cat* genes. Additionally, we observed that macrophages inoculated with inactivated *A. salmonicida* did not phagocytize the formalin-killed pathogen, and post-exposure to the bacterin, several apoptotic-like bodies were presented. These results suggest that inactivate *A. salmonicida* trigger a potent innate immunity modulated by the macrophage.

## Data Availability

All datasets generated for this study are included in the manuscript and/or the Supplementary Files.

## Ethics Statement

The animal experiments were performed in accordance with the guidelines of the Canadian Council on Animal Care and approved by Memorial University of Newfoundland's Institutional Animal Care Committee (protocols #17-01-JS; #17-02-JS).

## Author Contributions

MS-D and JS: conception and design of research. MS-D and AH: macrophage isolation. MS-D and SC: live and formalin-filled *A. salmonicida* preparation. MS-D, AH, and SC: performed experiments. MS-D, MR, and JS: interpreted results of experiments, edited and revised manuscript. MS-D and JS: prepared figures, drafted manuscript, and funding support. MS-D, AH, SC, MR, and JS: approved final version of manuscript.

### Conflict of Interest Statement

The authors declare that the research was conducted in the absence of any commercial or financial relationships that could be construed as a potential conflict of interest.
