# Supplementary material for: Aeromonas salmonicida subsp. salmonicida Early Infection and Immune Response of Atlantic Cod (Gadus morhua L.) Primary Macrophages
Source: Front Immunol. 2019 Jun 4;10:1237. doi: 10.3389/fimmu.2019.01237 (PMC6559310; doi:10.3389/fimmu.2019.01237)
Supplement: Supplementary file 1 [file Data_Sheet_1.docx]

**Supplementary information**

***Salmo salar* maintenance**

All the fish procedures were performed at the Dr. Joe Brown Aquatic Research Building (JBARB) at the Department of Ocean Sciences, Memorial University of Newfoundland, Canada. The animal protocols were approved by the Animal Care Committee at Memorial University (protocol #17-02-JS), based on the guidelines of the Canadian Council on Animal Care. Adult Atlantic salmon (1.8 ± 0.1 kg) were maintained under optimal conditions in 3,800 L tanks with 95-100% oxygen saturation, ambient photoperiod, with a flow-through seawater system using seawater that was filtered, UV-treated, and heated or chilled to maintain optimal water temperature (10-12°C). The biomass density was maintained in a range of 5-30 kg per cubic meter. The fish were fed 3 days per week at a level of 1% body weight per feeding time, using a commercial diet (Skretting, BC, Canada).

**Atlantic salmon primary macrophages isolation**

Atlantic salmon primary macrophages were isolated from head kidney in accordance with the protocol stablished by Smith et al. (2018) and Soto-Dávila et al. (2018) with modifications. Briefly, head kidney from 3 fish were removed and minced through 100 µm nylon sterile cell strainers (Fisher scientific) in isolation media (Leibovitz-15 medium, Gibco® supplemented with 1% penicillin/ streptomycin, 250 µg ml-1 heparin, and 0.1% FBS). Cells were centrifuged at 400 x g at 4°C in a 34/51% Percoll (GE Healthcare, Uppsala, Sweden) gradient, and primary macrophages obtained from the macrophage-enriched interface were washed twice with PBS. Total number of cells and viability were determined by using the Countness™ cell counter (Invitrogen), and trypan blue stain (0.4%; Invitrogen). Atlantic salmon primary macrophages were seeded in 6 well plates in a concentration of 1 x 10^7^ cells ml^-1^, and incubated at 15ºC for 24 h. After this period, cells were washed with PBS and incubated at 15°C for additional 5 days in 2 ml of culture media (L-15 supplemented with 1% penicillin/ streptomycin, 50 μM 2-mercaptoethanol, and 5% FBS) to ensure cell attachment until the infection.

- Smith, N.C., Christian, S.L., Taylor, R.G., Santander, J., and Rise, M.L. (2018). Immune modulatory properties of 6-gingerol and resveratrol in Atlantic salmon macrophages. Mol. Immunol. 95, 10-19.
- Soto-Dávila, M., Rise, M.L., and Santander, J. Antibacterial effects of cholecalciferol in Atlantic salmon (*Salmo salar*) primary macrophages infected with *Aeromonas salmonicida*. In: 8^th^ International Symposium on Aquatic Animal Health; 2018 Sept. 2-6; Charlottetown, Canada.
