# Supplementary material for: Aeromonas salmonicida subsp. salmonicida Early Infection and Immune Response of Atlantic Cod (Gadus morhua L.) Primary Macrophages
Source: Front Immunol. 2019 Jun 4;10:1237. doi: 10.3389/fimmu.2019.01237 (PMC6559310; doi:10.3389/fimmu.2019.01237)
Supplement: Supplementary file 2 [file Table_1.docx]

**Supplementary Table 1.** Ct values for Atlantic cod reference genes in primary macrophages after infection with *A. salmonicida* and inoculation with formalin-killed *A. salmonicida*.

| Treatment | Fish | EF-1α | Β-actin | 18S | Eif3 | 60S |
| --- | --- | --- | --- | --- | --- | --- |
| Control | **1** | 19.905 | 22.737 | 10.707 | 28.288 | 22.231 |
|  | **2** | 19.634 | 22.177 | 10.443 | 28.450 | 20.122 |
|  | **3** | 19.923 | 20.152 | 12.632 | 29.816 | 23.232 |
| 1 h post infection  live *A. salmonicida* | **1** | 19.749 | 23.261 | 10.932 | 28.177 | 22.509 |
|  | **2** | 19.741 | 22.781 | 11.556 | 29.256 | 22.349 |
|  | **3** | 19.614 | 20.569 | 13.036 | 30.889 | 21.359 |
| 2 h post infection  live *A. salmonicida* | **1** | 19.931 | 20.379 | 13.739 | 31.145 | 20.234 |
|  | **2** | 19.750 | 20.454 | 11.559 | 27.890 | 22.163 |
|  | **3** | 19.626 | 22.328 | 10.408 | 28.798 | 23.054 |
| 6 h post infection live *A. salmonicida* | **1** | 19.640 | 23.561 | 11.511 | 27.839 | 21.445 |
|  | **2** | 19.591 | 21.368 | 12.849 | 29.609 | 19.352 |
|  | **3** | 19.677 | 19.400 | 14.910 | 30.732 | 19.543 |
| 1 h post inoculation formalin killed *A. salmonicida* | **1** | 19.996 | 22.001 | 10.098 | 31.350 | 19.123 |
|  | **2** | 19.691 | 21.833 | 12.936 | 32.590 | 21.961 |
|  | **3** | 19.827 | 21.987 | 9.911 |  | 18.932 |
| 2 h post inoculation formalin killed *A. salmonicida* | **1** | 19.675 | 19.843 | 9.886 | 32.896 | 18.953 |
|  | **2** | 19.549 | 20.802 | 10.794 | 30.432 | 20.150 |
|  | **3** | 19.768 | 21.018 | 8.894 | 31.192 | 18.034 |
| 6 h post inoculation formalin killed *A. salmonicida* | **1** | 19.655 | 24.400 | 8.497 | 29.268 | 17.589 |
|  | **2** | 19.751 | 22.957 | 12.092 | 33.135 | 21.163 |
|  | **3** | 19.648 | 23.715 | 10.145 | 30.989 | 21.267 |

*****Each value represent the mean of technical replicates (n=3).
******geNorm M values were: 0.102 (*EF-1α*), 0.112 (*Eif3*), 0.138 (60S), 0.147 (*β-actin*), 0.190 (18S); M<0.15 for most stable genes [1].
*******BestKeeper values were: 0.101 (*EF-1α*), 1 (*Efi3*), 1.124 (*60S*), 1.175 (*18S*), 1.21 (*β-actin*); M<1 for most stable genes [2].

1. Vandesompele, J., De Preter, K., Pattyn, F., Poppe, B., Van Roy, N., De Paepe, A., and Speleman, F. (2002). Accurate normalization of real-time quantitative RT-PCR data by geometric averaging of multiple internal control genes. Genome Biol. 3, research0034-research0034.11.
2. Pfaffl, M., Tichopad, A., Prgomet, C., and Neuvians, T.P. (2004). Determination of stable housekeeping genes, differentially regulated target genes and sample integrity: BestKeeper – Excel-based tool using pair-wise correlations. Biotechnol. Lett. 26, 509-515.
