# Supplementary material for: Aeromonas salmonicida subsp. salmonicida Early Infection and Immune Response of Atlantic Cod (Gadus morhua L.) Primary Macrophages
Source: Front Immunol. 2019 Jun 4;10:1237. doi: 10.3389/fimmu.2019.01237 (PMC6559310; doi:10.3389/fimmu.2019.01237)
Supplement: Supplementary file 3 [file Image_1.pdf]

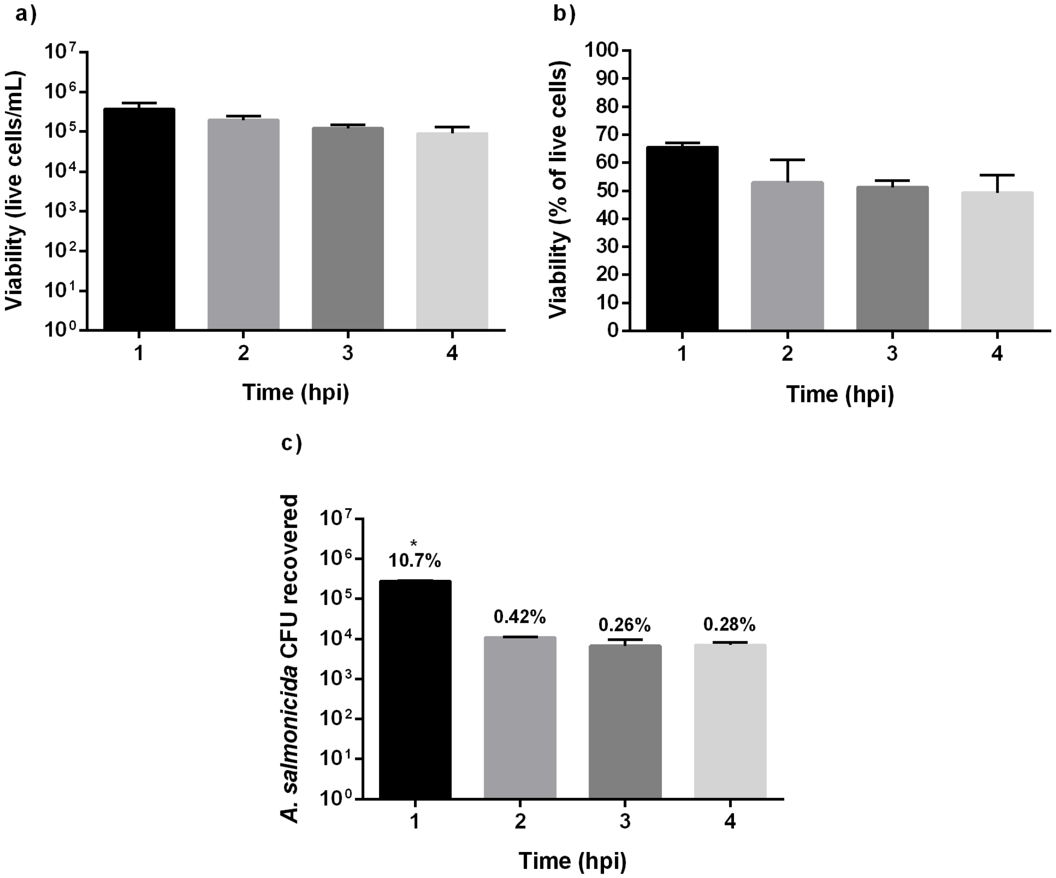

Supplementary Figure 1. Gentamicin exclusion assay in Atlantic salmon macrophages infected with *Aeromonas salmonicida* subsp. *salmonicida*. The figures show the number of live cells (a) and the percentage of viability (b), after 1, 2, 3 and 4 h post infection. The figure also shows the colony forming unit (c) recovered from cells during each time post infection. Each value is the mean ± S.E.M (n=3). Symbol (*) indicate statistical differences between each time post infection. Percentage show above bars indicate the total % of attach (1 h post infection) and invasion (2, 3 and 4 h post invasion) of *A. salmonicida* to Atlantic salmon macrophages, $P < 0.005$. Procedures for Atlantic salmon husbandry and macrophage extraction are described in the supplementary data.
