# Supplementary material for: Aeromonas salmonicida subsp. salmonicida Early Infection and Immune Response of Atlantic Cod (Gadus morhua L.) Primary Macrophages
Source: Front Immunol. 2019 Jun 4;10:1237. doi: 10.3389/fimmu.2019.01237 (PMC6559310; doi:10.3389/fimmu.2019.01237)
Supplement: Supplementary file 4 [file Image_2.pdf]

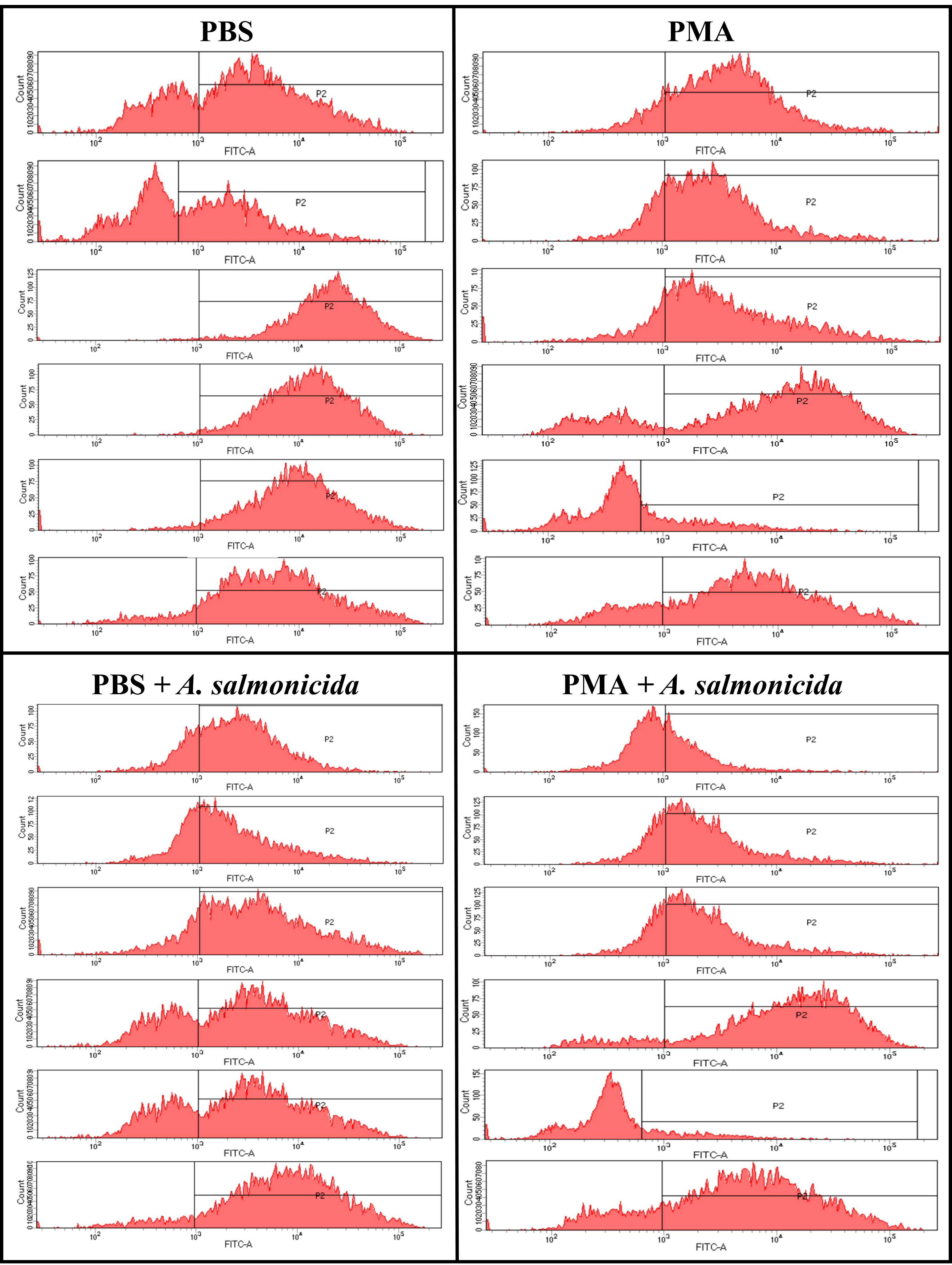
PBS
PMA
PBS + *A. salmonicida*
PMA + *A. salmonicida*
Count
FITC-A
P2
10^2
10^3
10^4
10^5

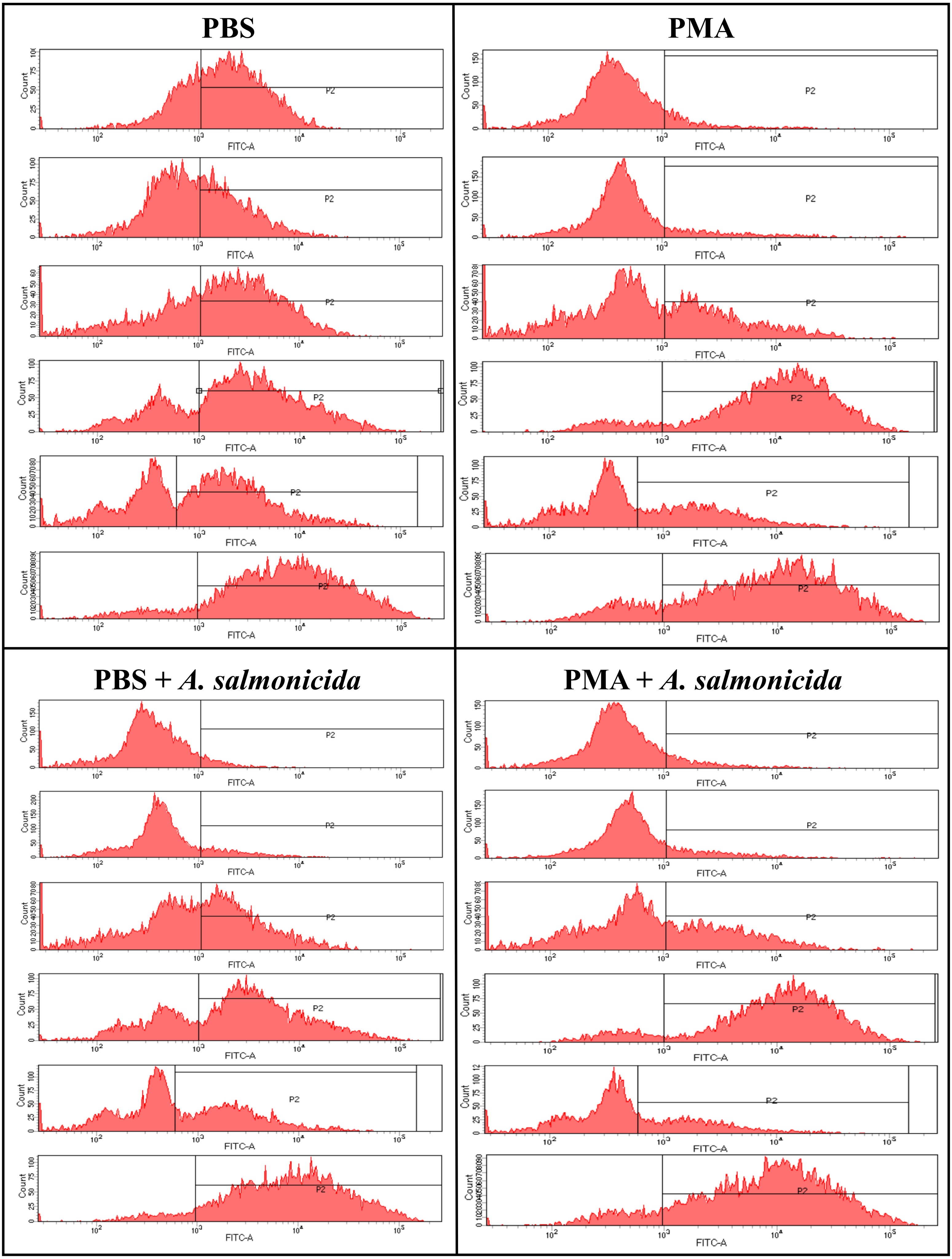

Supplementary Figure 2. Fluorescence histograms of ROS production in Atlantic cod primary macrophages 6 h post-inoculation with PBS (negative control), PMA (positive control), PBS + *A. salmonicida*, and PMA + *A. salmonicida* (n=6).
